# A new workflow for the effective curation of membrane permeability data from open ADME information

**DOI:** 10.1186/s13321-024-00826-z

**Published:** 2024-03-14

**Authors:** Tsuyoshi Esaki, Tomoki Yonezawa, Kazuyoshi Ikeda

**Affiliations:** 1 Faculty of Data Science, Shiga University, 1-1-1 Banba, Hikone, Shiga 522-8522 Japan; 2 Faculty of Culture and Information Science, Doshisha University, 1-3 Tatara Miyakodani, Kyotanabe, Kyoto 610-0394 Japan; 3 Faculty of Pharmacy, Keio University, 1-5-30 Shibakoen, Minato-ku, Tokyo, 105-8512 Japan; 4 HPC-and AI-Driven Drug Development Platform Division, RIKEN Center for Computational Science, 1-7-22 Suehiro-cho, Tsurumi-ku, Yokohama, Kanagawa 4230-0045 Japan

**Keywords:** Data curation, ADME, Membrane permeability, In silico model, KNIME workflow, ChEMBL

## Abstract

Membrane permeability is an in vitro parameter that represents the apparent permeability (Papp) of a compound, and is a key absorption, distribution, metabolism, and excretion parameter in drug development. Although the Caco-2 cell lines are the most used cell lines to measure Papp, other cell lines, such as the Madin-Darby Canine Kidney (MDCK), LLC-Pig Kidney 1 (LLC-PK1), and Ralph Russ Canine Kidney (RRCK) cell lines, can also be used to estimate Papp. Therefore, constructing in silico models for Papp estimation using the MDCK, LLC-PK1, and RRCK cell lines requires collecting extensive amounts of in vitro Papp data. An open database offers extensive measurements of various compounds covering a vast chemical space; however, concerns were reported on the use of data published in open databases without the appropriate accuracy and quality checks. Ensuring the quality of datasets for training in silico models is critical because artificial intelligence (AI, including deep learning) was used to develop models to predict various pharmacokinetic properties, and data quality affects the performance of these models. Hence, careful curation of the collected data is imperative. Herein, we developed a new workflow that supports automatic curation of Papp data measured in the MDCK, LLC-PK1, and RRCK cell lines collected from ChEMBL using KNIME. The workflow consisted of four main phases. Data were extracted from ChEMBL and filtered to identify the target protocols. A total of 1661 high-quality entries were retained after checking 436 articles. The workflow is freely available, can be updated, and has high reusability. Our study provides a novel approach for data quality analysis and accelerates the development of helpful in silico models for effective drug discovery. Scientific Contribution: The cost of building highly accurate predictive models can be significantly reduced by automating the collection of reliable measurement data. Our tool reduces the time and effort required for data collection and will enable researchers to focus on constructing high-performance in silico models for other types of analysis. To the best of our knowledge, no such tool is available in the literature.

**Supplementary Information:**

The online version contains supplementary material available at 10.1186/s13321-024-00826-z.

## Introduction

In the drug-discovery industry, the mean cost of R&D investments is 13 billion dollars, and new drug entries require 10–15 years to reach the market [1, 2]. Despite the tremendous efforts of researchers to discover new drugs, several drugs that used to be available in the market were dropped owing to their low efficacy or side effects. Discontinuation causes enormous losses to pharmaceutical companies and patients awaiting effective medicines. Therefore, effective methods to estimate pharmacokinetic parameters such as absorption, distribution, metabolism, and excretion (ADME) are required to accelerate drug discovery and reduce losses in the early stages of drug development.

Many pharmaceutical laboratories have demonstrated high-throughput screening (HTS) in the early stages and have collected massive in vitro experimental data on various pharmacokinetic parameters, including water solubility, metabolic intrinsic clearance, membrane permeability, and unbound fraction in the plasma protein. Experiments have enabled researchers to obtain vast amounts of data that are necessary to estimate compound features and select promising compounds over short time spans. Membrane permeability is an in vitro parameter that represents the apparent permeability (Papp) of a compound. It is one of the most essential ADME parameters since it strongly affects the absorption ratio of a compound and is necessary to determine dosage schedules.

The ADME parameters measured in vitro significantly vary, and Papp is affected by various experimental conditions, such as cell species, transporter overexpression, compound concentration, penetration direction, pH, and inhibitor usage [3]. Caco-2 cell lines generated from human colon cells are mainly used to estimate the intestinal absorption of administered compounds, and are the most used cell lines to measure Papp [4–6]. In silico models were developed to estimate the Papp measured in Caco-2 cell lines. Other cell lines, such as the Madin-Darby Canine Kidney (MDCK), LLC-Pig Kidney 1 (LLC-PK1), and Ralph Russ Canine Kidney (RRCK) cell lines, were also used to measure Papp. MDCK is derived from the canine kidney and does not express human transporters (such as multi-drug resistance 1 (MDR-1)) [7, 8]. Therefore, the MDCK cell lines were used to quantitatively estimate the efflux ratio of overexpressed human MDR1 transporter cells compared with that of wild-type MDCK cells. LLC-PK1 is a cell line developed from pig kidney and expresses endogenous transporters of nonhuman origin [9]. The RRCK cell lines originated from the MDCK cell lines. Canine MDR-1 in MDCK interferes with permeability and transporter studies; thus, the data obtained from such studies may have low reliability. The cell line was developed from a subpopulation of low-efflux cells from MDCKII-WT; thus, this cell line is also called the MDCKII-LE (low efflux) cell line [10]. RRCK cells are usually used to estimate penetration through the blood–brain barrier by using overexpressed human MDR-1 transporter cells. One of the merits of these cell lines compared with Caco-2 cell lines is their short preparation time after seeding. Caco-2 cells require approximately 3–4 weeks to prepare; in contrast, MDCK, LLC-PK1, and RRCK cells can be prepared within 4–5 days. The Papp measured in these cell lines can effectively estimate membrane permeability. Therefore, constructing in silico models to estimate Papp using the MDCK, LLC-PK1, and RRCK cell lines is necessary.

Estimating Papp is a critical issue over the last decade [3]. In silico methods that enable the application of various features from chemical structures alone are required to estimate the Papp parameters of new entries. Several in silico models to predict Papp measured in the Caco-2 cell lines were developed [11–20]; however, the models constructed to estimate Papp obtained from other cell lines are usually referenced to Caco-2 models [11, 16]. These models are constructed on artificial intelligence (AI) including machine learning using massive datasets, and provide new approaches to evaluate the efficacy and safety of drug candidates in the early drug discovery stage [21].

Collecting large amounts of in vitro Papp experimental data to construct in silico models is extremely important. Several laboratories such as bio-ventures and academic laboratories have difficulty in collecting sufficient measurements for model construction since HTS requires actual compounds, experimental instruments, and considerable time and costs. Furthermore, compounds measured in one laboratory are likely to have similar substructures since these compounds are generally measured in the same project (the same laboratory). Developing models to predict drug properties using compounds with similar chemical spaces is practical. However, these models may encounter difficulties in predicting the properties of compounds in different chemical spaces. Generally, in silico models are used in the early stages of drug discovery to predict the characteristics of new entries in a vast chemical space. Therefore, a collection of various compounds is necessary to construct high-precision and versatile in silico models.

An open database helps collect massive measurements of various compounds covering a vast chemical space. The ChEMBL database is a manually curated database containing a large amount of bioactivity and pharmacokinetic experimental data [22, 23]. The use of this database for data collection helps construct in silico models. However, concerns were reported on the use of data published in open databases without checking their accuracy and quality [24]. Furthermore, curation generally incurs considerable cost and time [25]. Tiikkainen et al. reported the error rate of several bioactivity databases, including ChEMBL [26], and reported that humans performed data checks that occasionally included incorrect data owing to human error. Ensuring accuracy when collecting experimental data divided into cell lines is challenging, and the presence of miscellaneous data reduces the performance of the constructed predictive models [27, 28]. Therefore, the data curation of experimental protocols is important [29].

Data curation is performed in cheminformatics to check compound structures, such as mixtures and tautomers [30, 31]. Manual curation is costly and time consuming, and maintaining up-to-date datasets by periodically checking the rapidly growing volume of publications is difficult. Curation in cheminformatics generally focuses on chemical-structure cleaning and data standardization; therefore, tools to support their procedures were developed to overcome these hurdles [31, 32]. The applications of AI have recently progressed to data preparation and have contributed to data collection in materials informatics [33]. However, only curating chemical structures is insufficient to prepare a dataset to predict ADME properties, including Papp. Checking experimental protocols with expert knowledge from pharmacokinetics experiments is also necessary because such experiments feature various measurement methods related to the aim of the study [34]. However, these various protocols produce noise during model construction. Collecting experimental data measured using unified protocols or units is helpful to construct in silico models. Careful collection and curation of published data plays an essential role in constructing highly accurate in silico Papp models divided into cell lines.

Despite the extensive efforts of researchers, manual curation may cause inaccurate data collection. Furthermore, informatics and experimental researchers must be involved during data curation to check the Papp experimental data. We wish to highlight the importance of data curation under experimental conditions. This study focused on identifying specific issues related to data curation and improving their efficiency. We developed an automatic support tool to curate the Papp data measured in the MDCK, LLC-PK1, or RRCK cell lines collected from ChEMBL. The entries measured membrane permeability with MDCK, LLC-PK1 or RRCK not involving overexpressing cells and apical to basolateral direction were retained. The protocols are the most generally used. The values measured using these protocols are useful to estimate the permeability of the compounds. KNIME is a workflow platform that visualizes analytical processes such as machine learning [35]. It was employed to develop a workflow for data curation in this study. KNIME was used to curate the chemical structure [32] and predict the properties of drugs [36–38], including ADME [39, 40] owing to its user-friendly open-source platform and customizable module. In our study, experimental ADME data downloaded from ChEMBL were imported into KNIME, and descriptions of the experiment were automatically checked at the nodes. The data were subsequently exported in the comma-separated values (CSV) and structure data file (SDF) formats after unifying the experimental units (Additional files 2, 3, 4, 5, 6).

Researchers have pointed out the importance of providing and sharing reusable protocols to avoid data errors and problems experienced by others in the past [41]. The main advantages of our tool are as follows: (1) it uses a graphical interface that allows all researchers to visualize the data curation process; (2) it provides an easy-to-use and reproducible protocol for data extraction; (3) it presents a reusable general pipeline that integrates different data curation procedures; and (4) it outlines the steps for manual literature curation to confirm the experimental findings and can be extended to further refine manual curation.

The proposed workflow enabled us to effectively curate and collect Papp data measured in the MDCK, LLC-PK1, and RRCK cell lines. Furthermore, the cell lines in KNIME can be modified to obtain Papp data that were measured in the Caco-2 cell lines. Researchers can also adapt other ADME parameter-modification nodes in the workflow. We believe that our workflow can be used to collect various Papp data and support the construction of highly accurate in silico prediction models to accelerate drug discovery.

## Results

**Fig. 1:**
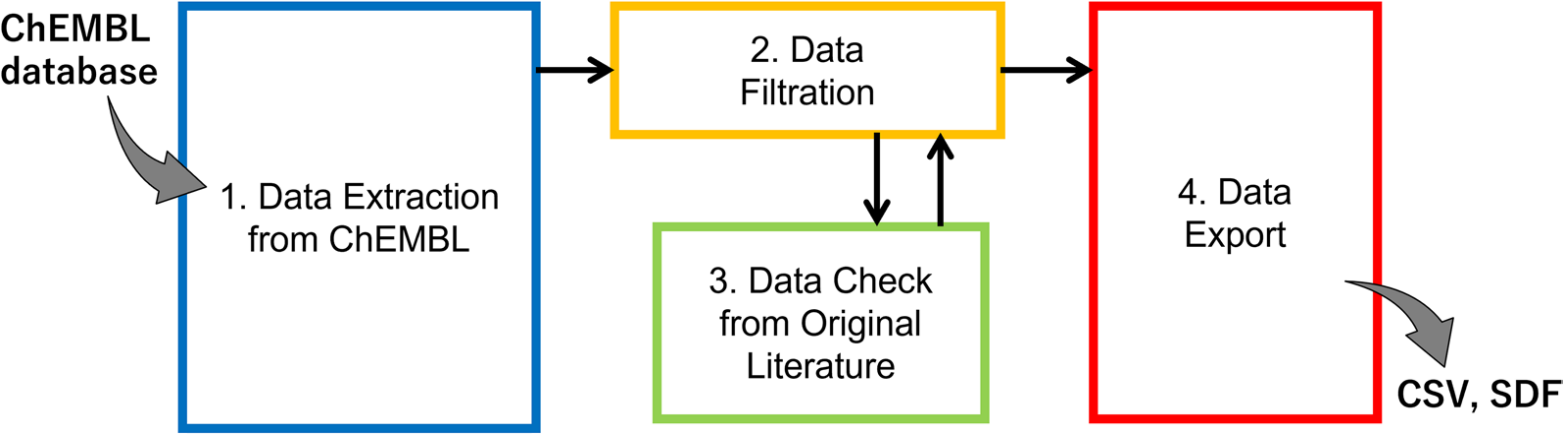
Scheme of the data curation workflow: Each box represents the procedure phase in the Results

This section provides general information on the development of our workflow (Fig. 1) and the principles behind each step. Our workflow consists of four main phases: (1) data extraction from ChEMBL, (2) data filtration, (3) data checking against the original literature, and (4) data export. The details of each step are provided in the Additional file 1: Figs. S1–S4.

### Phase 1. Data extraction from ChEMBL

The ChEMBL SQL dump (ver. 33) was downloaded from the ChEMBL website (https://ftp.ebi.ac.uk/pub/databases/chembl/ChEMBLdb/releases/chembl_33/) [23] and imported into KNIME using the SQLite data source connector. Data were collected from seven tables: activities, assays, docs, molecule_dictionary, compound_properties, compound_records, and compound_structure.

Activity data in the activities table were obtained by collecting the experimental Papp data and filtering the entries using the keyword “standard_type LIKE Papp.” Data were filtered using the terms “description LIKE %permeability%” to link them outerly to the activities table by assay_id (Table 1, Step 1). Information on the original articles for each entry (the activity value endpoint) was linked to the docs table in the source information. The docs table was extracted to obtain literature information in KNIME. The collected entries were linked to the connected data (activities and assays) by doc_id (Table 1, Step 2). Chembl_id was used to identify the compounds collected from the molecule_dictionary table. The chembl_id values in molecule_dictionary were added to the connected data (activities, assays, and docs) using molregno (Table 1, Step 3). Compound properties, such as molecular weight (MW) and formula, were collected to identify compounds with the same experimental values. These properties were obtained from the compound_properties table. The obtained properties were also inner joined to the connected tables (activities, assays, docs, and molecule_dictionary) using molregno (Table 1, Step 4). Several molregno entries included multiple compound names such as synonyms or trade names. Therefore, the group-by function was performed for molregno in compound_records to obtain a one-to-one correspondence between values. The molregno in this table and the connected data were used as critical columns to merge the two tables (Table 1, Step 5). Finally, the compound_structure table was extracted to obtain compound structure information. The molregno values in the compound_structure table were used to join the columns, and the table was merged into a connected table (Table 1, Step 6).

**Table 1:** Summary of entry numbers in a data extraction session.

Step	Table 1	Table 2	Joining columns (Join mode)	Retained entries
1	Activities	Assays	assay_id (Full outer join)	22,689
2	Combined activities and assays	Docs	doc_id (Inner join)	22,614
3	Combined activities, assays, and docs	Molecule_dictionary	molregno (Inner join)	16,847
4	Combined activities, assays, docs, and molecule_dictionary	Compound_property	molregno (Inner join)	16,847
5	Combined activities, assays, docs, molecule_dictionary, and compound_properties	Compound_records	molregno (Inner join)	16,847
6	Combined activities, assays, docs, molecule_dictionary, compound_properties, and compound_records	Compound_structures	molregno (Inner join)	16,845

Table 1 shows the table obtained by combining the previous steps, and Table 2 shows the new table. The join mode in join columns is used in KNIME

Data extraction from ChEMBL and conversion of the table to a usable format were demonstrated using the DB Table Selector and DB Reader nodes in KNIME. Each step requires a large amount of memory because the DB Table Selector accesses the database and collects many entries. To avoid high loads, we processed each combination after completing the previous steps. Following these procedures, we collected a total of 16,845 entries. In Step 6, one entry whose molecular formula was Na_3_O_4_V (activity_id = 19364594) was rejected since it was not included in the molecular_structures table.

The workflow is shown in Additional file 1: Fig. S1, and details of the collected items and number of entries in each table are listed in Additional file 1: Table S1.

**Table 2:** Summary of the results of each process

Step	Procedure	Retained entries	Number of columns
1	Remove no-value, relation, and unit	15,470	28
2	Remove different units	15,433	28
Through Phase (3)			
3	Merge curated entries	2486	29
4	Remove columns not used for analysis	2486	13

### Phase 2. Data filtration

The extracted entries were filtered based on the experimental information. The columns used in the table merge (doc_id and assay_type) were removed since they were not used after the previous procedures.

This phase consists of two steps. First, the entries were filtered using the experimental data and protocols. The experimental results were necessary to analyze data including QSAR. Since, several data points had no experimental values, relations, or units, these entries could not be used for the data analysis. Therefore, these entries were removed in this first step (the number of retained entries is shown in Table 2, Step 1). The Papp measurement data were collected using this workflow. Entries unrelated to the Papp measurements (entries with “%” in standard_units) were removed (Table 2, Step 2).

The process was separated into the next node and the Data Check Phase (Phase 3). Subsequently, the filtered and checked entries were merged by reading the original articles. Entries in the data check phase were confirmed or fixed to obtain the correct information (details of the data checks are explained in Phase 3).

The merged table contained three new columns: curated_value, curated_unit, and frag (Table 2, Step 3). The columns collected in this phase were confirmed, and unnecessary columns that were not used for data analysis were removed (Table 2, Step 4).

The changes in the entries are summarized in Table 2, and the details of this step are shown in Additional file 1: Fig. S2.

### Phase 3. Data check from original literature

Occasionally, several anomalies were observed in the filtered data. For example, unusually large Papp values, such as 119,000,000, 96,000,000, and 91,000,000, were obtained in some instances. These extremely high values are potentially incorrect, and the workflow should be modified to collect values with units referring to the units, and the original literature depending on the case. The units of Papp in the entries collected from the database also varied as follows: cm s^−1^, nm s^−1^, cm min^−1^, 10e^−6^ cm s^−1^, nm/s, and ucm/s (Additional file 1: Table S2). Therefore, unification of these units is essential for data curation. The unit representing Papp measured in terms of membrane permeability is generally 10^−6^ cm/sec. A description is also necessary to verify the experimental protocol (such as the cell line and penetration direction) to confirm the correctness of the measurements. Almost all entries contained information on the cell line, direction, and transporter overexpression. However, ambiguous descriptions of the experimental protocols were sometimes included in the results (such as “apparent permeability of the compound,” “apparent permeability by cell based assay,” and “permeability was determined”).

Furthermore, the permeability assays reported two protocols for the flow direction of compounds: from apical to basolateral and from basolateral to apical. The former (from apical to basolateral) is generally used for Papp assays and the latter (from basolateral to apical) is used to study the influence of transporters. Many entries contained this information in their description, but some did not. These data appear to have been generated from indistinct descriptions in the articles or a lack of expert knowledge. The columns assay_organism and assay_cell_type helped to determine the experimental protocols. *Homo sapiens* in the assay_organism column represents the use of the Caco-2 cell lines in assay_cell_type. Similarly, *Canis lupus* indicates the use of MDCK cells. However, several entries showed different information. For example, some entries with *Homo sapiens* in assay_organism had MDCK in assay_cell_type. These data indicate the permeability of MDCK cells overexpressing human p-glycoprotein. In this case, the assay_organism column shows the organism in the assay cell or overexpressing transporters. Ambiguous entries are often problematic when determining whether they should be removed using only the information imported from ChEMBL. Therefore, reading the original articles and confirming the experimental values, units, and protocols is necessary to collect faithful experimental data on Papp across the apical to basolateral direction in MDCK, LLC-PK1, and RRCK cells.

The original articles in the entries obtained during this phase were checked. Entries including Papp data measured in the MDCK, LLC-PK1, and RRCK cell lines were identified. Most of the entries included articles with only one measurement (Additional file 1: Table S3). A total of 436 articles (the majority of which were from *ACS. Med. Chem. Lett.*, *J. Med. Chem.*, and *Bioorg. Med. Chem. Lett.*) were checked, and the experimental measurements, units, and protocols were reviewed. Data obtained through ambiguous protocols, data collected from basolateral to apical, data using transporter-overexpressing cells, data measured in the presence of inhibitors, and average data of different directions (average of apical to basolateral and basolateral to apical) were rejected. The number of entries decreased from 15,433 to 2486 because most entries that reached Phase 3 were Papp data measured in Caco-2 cells (7713 entries in 15,433) from the assay_cell_type column. Other reasons for rejection included measurements using different protocols (such as permutation measurements from basolateral to apical, different pH values, or overexpressed MDR-1) (Table 3, original data is Additional file shared as a curated output table). The collected entries were examined and curated using these procedures. The original articles were checked to determine whether the cell lines overexpressed MDR1. However, we construct nodes to filter whether the entries were measured using required protocols and cell lines by description ("not including MDR, Pgp or express" and "including like from apical ") and assay_cell_type ("MDCK", "LLC-PK1" or "RRCK or MDCKII-LE") for more usability. A new column was made (manually_curated for output data) to distinguish the manually curated data and filtered data using these new nodes.

**Table 3:** Summary of the reasons for rejecting entries obtained through data curation using the original articles

Rejection reason	Number of unique entries	Number of unique chembl_id values	Number of unique doc_id values	Number of unique assay_id values	Number of entries of autocuration in curated_by columns in the assay table
Ambiguous cell lines from the original article	350	323	86	97	343
Average of apical to basolateral and basolateral to apical velocities	91	91	8	8	91
Different cell lines (such as Caco-2, 2/4/A1, PAMPA, and Lucifer Yellow Permeability)	152	108	24	36	129
From basolateral to apical (in description)	372	310	67	88	317
Expressing MDR1 (p-glycoprotein) and BCRP	731	667	119	133	596
Different pH (pH 5.5 or 6.5)	4	2	1	2	4
Measurements of efflux ratio	3	3	1	1	0

Several duplicates were obtained

The retained entries were returned to the node in Phase 2 and merged with the original tables. The details of Phase 3 are shown in Additional file 1: Fig. S3, and the curated article information is also provided in the Supporting Information.

ChEMBL data are updated multiple times each year; however, maintaining updated datasets by periodically checking them is difficult. Therefore, nodes were prepared to store the checked information from the original articles, and a curated memo was generated during this phase. Initially, entries that were unchecked were collected and compared with previously checked entries. Subsequently, entries requiring checking were outputted from the KNIME workflow to check against the original data. Finally, the articles in these entries were reviewed and the checked results were imported into KNIME and merged with the previously checked entries.

### Phase 4. Data export

The entries retained through the protocol check and filters in Phase 3 are helpful for various analyses, including similarity analysis and machine learning. Therefore, they were exported to formats that could be used by researchers. Given the variety of tools available, the CSV and SDF formats are convenient. In total, 13 sets of information for SDF and 12 sets of information for CSV were considered useful: activity_id, pubmed_id, molregno, chembl_id, canonical_smiles, molfile (for SDF), full_mw, full_molformula, standard_relation, curated_value, curated_units, curated_cell_line (information on cell lines), and manually_curated. The output date were attached to the file names to confirm the update of these files. The output data are available in the Supporting Information, and details of this step are shown in Additional file 1: Fig. S4.

## Discussion

### Distribution of the curated datasets

**Fig. 2:**
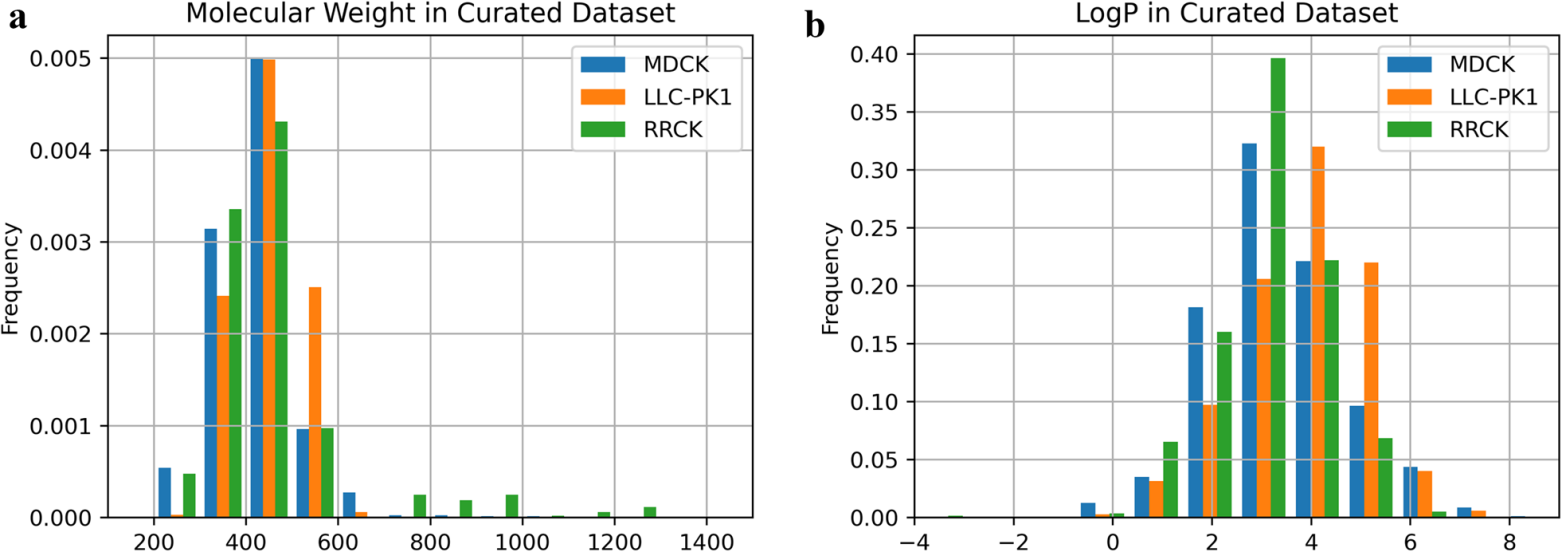
Distribution of compounds in the curated datasets: The left (**a**) and right (**b**) figures show the molecular weight and ClogP of each compound measured using three cell lines, respectively. The blue, orange, green bars represent the data of MDCK, LLC-PK1 and RRCK, respectively. It is necessary to compare the properties of compounds (especially molecular weight and ClogP) to consider the drug-likeness of compounds

The numbers of output entries for MDCK, LLC-PK1, and RRCK (including LE-MDCKII) were 1,324 (875 compounds), 422 (341 compounds), and 739 (517 compounds), respectively. Furthermore, manually curated output entries for MDCK, LLC-PK1, and RRCK (including LE-MDCKII) were 795 (738 compounds), 336 (322 compounds), and 530 (515 compounds), respectively. The distributions of the MW and ClogP of the unique compounds in the manually curated datasets are shown in Fig. 2. The plots show few differences in the distribution of these datasets. The MW and ClogP of the LLC-PK1 dataset were slightly higher than those of the other datasets. The average MW and ClogP of the RRCK dataset were lower than those of the other datasets (Table 4). However, only a few differences were observed between the datasets. This finding implies that these measurements may have different projects and targets in these datasets. The data contain valuable entries for correlation analysis and the construction of predictive models using machine learning.

**Table 4:** Summary of the average compound features in each manually curated dataset

Feature	MDCK	LLC-PK1	RRCK
Papp	12.19 (0.00–105.00)	15.43 (0.50–75.20)	11.64 (0.10–53.00)
MW	426.31 (208.26–1001.15)	448.86 (259.35–612.88)	418.88 (216.22–1202.64)
ClogP	3.39 (− 0.49 to 8.50)	3.86 (− 0.54 to 7.35)	2.87 (− 3.61 to 5.56)

MW and logP calculated by RDKit (ClogP) were calculated using unique compounds in each dataset

The values in parentheses represent the minimum and maximum values

However, the relationships between the compound features and Papp values were not clarified (Additional file 1: Fig. S5). In the manually curated data, the number of unique compounds included in the MDCK and LLC-PK1 measurements was 1131. The number of unique compounds in the LLC-PK1 and RRCK datasets was 709, while that in the MDCK and RRCK datasets was 1168. The number of compounds included in all curated datasets was 1504. Few compounds were included in multiple datasets.

### Differences between data filtered according to the ChEMBL entries and data curated in the original literature

The Papp data were collected using the ChEMBL information filter alone, without a literature check, and compared with curated datasets to check the effect of data curation.

Initially, the Papp data measured from MDCK cells was filtered from the output table by applying the curated_cell_line = “MDCK” and manually_curated = blank. A total of 529 entries were collected using these keywords (“filtered MDCK dataset”). Meanwhile, the number of manually curated MDCK entries that was collected through literature review were 795 (select curated_cell_line = “MDCK” and manually_curated = “Manually Curated” in the output csv file). In the filtered MDCK dataset, several incorrect entries of MDCK measurements were observed as follows: “collected using overexpressed MDR1” and “average of the two different direction data” referred to original articles. Entries that were not abnormal in the ChEMBL information but contained no information in the original articles were identified and removed as ambiguous entries. The entries were included in the filtered MDCK dataset but were removed from the manually curated MDCK dataset (Table 5). The entries in Table 5 provide examples of the data. In ChEMBL2, the Papp value of activity_id 18947687 was over five times higher than that that of activity_id 15456529. The detailed protocols of activity_id 18947687 (including the cell lines) were not found in the original literature. The data seemed to be measured using different protocols, although we stress that this finding is simply our speculation because we found no solid evidence of this practice. Thus, merging these two entries for the development of predictive models is inappropriate.

**Table 5:** Examples of compounds with retained and removed entries in MDCK and RRCK

Cell line	chembl_id	Compound	activity_id	Papp (10^–6^ cm/s)	Curation
MDCK	CHEMBL2		15,456,529	4.32	Retained
			18,947,687	27.62	Removed in manually check (not found)
RRCK	CHEMBL601719		13,941,269	0.8	Retained
			18,761,142	0.28	Removed (MDR AB)

The chemical structures were drawn using ChemDraw (ver. 22.2.0: PerkinElmer. Informatics)

Subsequently, the number of filtered LLC-PK1 entries (curated_cell_line = “LLC-PK1” and manually_curated = blank in the output csv file) was 86 (“filtered LLC-PK1 dataset”). The number of manually curated LLC-PK1 entries that was collected through literature review was 336 (select curated_cell_line = “LLC-PK1” and manually_curated = “Manually Curated” in the output csv file).

Finally, the number of filtered RRCK entries (curated_cell_line = “RRCK” and manually_curated = blank in the output csv file) was 210 (“filtered RRCK dataset”). The number of manually curated RRCK entries that was collected through literature review was 530 (select curated_cell_line = “RRCK” and “LE-MDCK2” and manually_curated = “Manually Curated” in the output csv file). The entries in Table 5 provide examples of the data. The Papp value of activity_id 18761142 was lower than that of activity_id 13941269, and the entry with the lower value was removed because of the following reasons. The removed entry (activity_id 18,761,142) was measured using MDR AB, the details of which were not mentioned in the original article. The data show the ratio of MDR AB to BA; here, the values appeared to be measured to study the efflux effects of the MDR transporter. However, the efflux ability value was not collected in our case; thus, this entry can reasonably be removed to avoid noise during data analysis.

Our workflow enabled us to collect data that were similar to those we desired. However, ambiguous and improper entries were present in the dataset. Thus, the relevant articles must be carefully read, and a workflow must be developed to more simplify data curation to improve the quality of the dataset. Currently, large amounts of time spent reading articles and expert knowledge are required to make sound experimental decisions. Our workflow was helpful to find ambiguous and improper data; thus, it can be potentially used to curate experimental protocols and collect high-quality datasets.

## Conclusions

We present a new tool to support the collection of experimental Papp data from the ChEMBL database for drug discovery. The collection of data that satisfies quality and quantity requirements is essential to construct in silico models with high predictive ability. Moreover, the collection of large amounts of data from open databases requires considerable research effort and time. Although the workflow used ChEMBL data as inputs, it is also applicable to open ADME data from any literature by using document-specific IDs (such as PMID and DOI) linked to experimental data points. Data collection requires careful curation because pharmaceutical data (including Papp) may include ambiguous entries measured using different cell lines or cells overexpressing transporters. Thus, checking and collecting the measured data using a single protocol is necessary. Furthermore, there were several entries with the unit "nM/s" despite "nm/s” in the original articles. The "M" is generally used as molar, and using the unit as meter may cause confusion. Checking this unit is critical in this study. Reducing the effort required for data collection is essential to accelerate data collection. Accelerated data collection with high-quality measurements enables the construction of high-performance predictive models. Therefore, we constructed a tool that supports data collection and curation using the KNIME workflow. The advantage of using KNIME is that it is a freely available tool. It can be easily used by researchers, such as informatics and non-informatics scientists. Therefore, our workflow offers the possibility of user customization. The modification of several nodes allowed our workflow to collect Papp measurements from other cell lines (such as Caco-2 and PAMPA). It is also helpful for collecting various data to support ADME predictions.

The quality of datasets for training in silico models is critical because AI (including deep learning) was used to develop models to predict various pharmacokinetic properties since quality effects the model’s performance owing to overfitting and limitations in its range of applications. Thus, the careful curation of collected data has become increasingly important.

The applications of AI have recently progressed to include property prediction and dataset preparation. The usage of AI may enable easy data curation and could advance data collection in the future. However, filtering experimental protocols using AI is difficult since the pattern of experimental descriptions varies. Data curation requires human resources and an effective combination of human efforts and AI. Checking the data quality of ChEMBL using a visualizable tool such as the KNIME workflow constructed in this study is necessary to maximize these combined effects.

The workflow developed in this study is available in the Supporting Information. Our study provides an opportunity for researchers to analyze data quality and accelerate the development of helpful in silico models. Our future research will continue to develop curation workflows to collect large high-quality ADME datasets and construct high-accuracy models to accelerate effective drug discovery.

## Methods

### Data collection

The Papp experimental data were collected from the ChEMBL database (ver. 33) [23], which manually curates and stores enormous amounts of experimental data related to physicochemistry and pharmacology. The database was downloaded from the website https://chembl.dl [42].

### KNIME workflow

KNIME (ver. 5.2) [35] is an open-source data analysis software (https://www.knime.com/) used to develop the workflow for Papp data curation. The nodes were exploited using the KNIME Analysis Platform and Schrodinger Extensions for KNIME.

### Feature calculation

The Molecular Weight (MW) and logP (ClogP) of the obtained compounds were calculated using RDKit (ver. 2022.09.5) to compare the distribution of compounds in the datasets [43]. RDKit is a cheminformatics tool that was used in Python (ver. 3.9.13) with a Jupyter Notebook.

### Supplementary Information

**Additional file 1.** Supporting information. **Table S1.** Keywords used for data extraction from the ChEMBL database. **Table S2.** Units collected from the database. **Table S3.** Number of measurements for each article. **Fig. S1.** Schema of phase 1: data extraction from ChEMBL. **Fig. S2.** Schema of phase 2: data filtration. **Fig. S3.** Schema of phase 3: data checking from the original literature. **Fig. S4.** Schema of phase 4: data export. **Fig. S5.** Relationships between features (molecular weight (MW) and logP calculated by RDKit (ClogP) of manually curated compounds.

**Additional file 2.** KNIME workflow.

**Additional file 3.** Output SDF.

**Additional file 4.** Output CSV file.

**Additional file 5.** Curated entry list (CSV).

**Additional file 6.** Curated output list (CSV).

## Abbreviations

ADME: Absorption, distribution, metabolism, and excretion
AI: Artificial intelligence
HTS: High-throughput screening
LLC-PK1: LLC-Pig Kidney 1
MDCK: Madin-Darby Canine Kidney
Papp: Apparent permeability
RRCK: Ralph Russ Canine Kidney
SDF: Structure data file
