# Supplementary material for: A new workflow for the effective curation of membrane permeability data from open ADME information
Source: J Cheminform. 2024 Mar 14;16:30. doi: 10.1186/s13321-024-00826-z (PMC10938840; doi:10.1186/s13321-024-00826-z)
Supplement: Supplementary file 1 — Additional file 1. Supporting information. Table S1. Keywords used for data extraction from the ChEMBL database. Table S2. Units collected from the database. Table S3. Number of measurements for each article. Fig. S1. Schema of phase 1: data extraction from ChEMBL. Fig. S2. Schema of phase 2: data filtration. Fig. S3. Schema of phase 3: data checking from the original literature. Fig. S4. Schema of phase 4: data export. Fig. S5. Relationships between features (molecular weight (MW) and logP calculated by RDKit (ClogP) of manually curated compounds. [file 13321_2024_826_MOESM1_ESM.docx]

Additional file

A New Workflow for the Effective Curation of Membrane Permeability Data from Open ADME Information

Tsuyoshi Esaki^1,2*^, Tomoki Yonezawa^3^, Kazuyoshi Ikeda^3,4^

1. Graduate School of Data Science, Shiga University, 1-1-1 Banba, Hikone, Shiga 522-8522, Japan
2. Faculty of Culture and Information Science, Doshisha University, 1-3 Tatara Miyakodani, Kyotanabe, Kyoto 610-0394, Japan
3. Faculty of Pharmacy, Keio University, 1-5-30 Shibakoen, Minato-ku, Tokyo 105-8512, Japan
4. HPC-and AI-driven Drug Development Platform Division, RIKEN Center for Computational Science, 1-7-22 Suehiro-cho, Tsurumi-ku, Yokohama, Kanagawa 4230-0045, Japan

Table of Contents

1. Table S1: Keywords used for data extraction from the ChEMBL database S2
2. Table S2: Units collected from the database S3
3. Table S3: Number of measurements for each article S4
4. Fig S1: Schema of phase 1: data extraction from ChEMBL S5
5. Fig S2: Schema of phase 2: data filtration S6
6. Fig S3: Schema of phase 3: data checking from the original literature S7
7. Fig S4: Schema of phase 4: data export S8
8. Fig S5. Relationships between features (molecular weight (MW) and logP calculated by RDKit (ClogP) of manually curated compounds S9

Table S1. Keywords used for data extraction from the ChEMBL database.

| Collected table | Collected column | Filter word | Number of collected data |
| --- | --- | --- | --- |
| activities | activity_id  assay_id  molregno  standard_relation  standard_value  standard_units  standard_type | standard_type LIKE Papp | 16,922 |
| assays | assay_id  doc_id  description  assay_type  assay_organism  assay_tissue  assay_cell_type  curated_by | description LIKE %permeability% | 8,944 |
| docs | doc_id  journal  year  volume  issue  first_page  last_page  pubmed_id |  | 886,630 |
| molecule_dictionary | molregno  chembl_id |  | 2,399,743 |
| compound_property | molregno  full_mw  full_molformula |  | 2,376,273 |
| compound_record | molregno  compound_name | groupby in molregno | 2,312,287 |
| compound_structure | molregno  molfile  canonical_smiles |  | 2,372,674 |

Table S2. Units collected from the database.

| Units | Number of entries | Units | Number of entries |
| --- | --- | --- | --- |
| 10'-6 cm/s | 4550 | 10'-6cm/s | 9 |
| ucm/s | 4244 | 10'-6cm/s2 | 8 |
| 10^-6 cm/s | 2807 | cm min-1 | 8 |
| nm/s | 2417 | 10^-5 cm/s | 7 |
| 10'6cm/s | 183 | ucm | 6 |
| cm s-1 | 131 | umol/L/s | 6 |
| 10'-7 cm/s | 112 | um/s | 5 |
| cm/s | 102 | 10'-3cm/min | 4 |
| 10'-5cm/s | 91 | 10'-8m/s | 4 |
| cm/s * 10E6 | 90 | 10^-6/cm | 4 |
| uL/hr/cm2 | 70 | cm'-6/s | 4 |
| 10'-5 cm/s | 63 | nM s-1 | 4 |
| 10^6cm/s | 63 | 10-6 cm s-1 | 3 |
| nM/s | 61 | 10^-5 cm/min | 3 |
| 10'-4 cm/s | 50 | nmol/sec | 3 |
| 10^7cm/s | 50 | 10'-6cm | 2 |
| 10^-7 cm/s | 43 | 10'-6m/s | 2 |
| 10^-8cm/s | 41 | uM | 2 |
| 10^-5cm/s | 30 | 10'-3cm/s | 1 |
| 10'-6/cm | 26 | 10'-6/s | 1 |
| nm s-1 | 25 | 10'-9cm/s | 1 |
| 10e-6 cm s-1 | 23 | 10^-6cm2/s | 1 |
| 10'-8cm/s | 20 | cm2/sec | 1 |
| 10'-6nanometer/s | 16 | microg/cm2 | 1 |
| 10'-2 cm/s | 11 | microg/cm3 | 1 |
| 10'-6cm/min | 11 | nanometer/sec | 1 |
| 10-6 cm/s | 11 |  |  |

Table S3. Number of measurements for each article.

| Number of measurements | Frequency of articles | Cumulative frequency of articles | Cumulative ratio |
| --- | --- | --- | --- |
| 1 | 77 | 77 | 0.1754 |
| 2 | 72 | 149 | 0.3394 |
| 3 | 32 | 181 | 0.4123 |
| 4 | 44 | 225 | 0.5125 |
| 5 | 13 | 238 | 0.5421 |
| 6 | 28 | 266 | 0.6059 |
| 7 | 16 | 282 | 0.6424 |
| 8 | 21 | 303 | 0.6902 |
| 9 | 5 | 308 | 0.7016 |
| 10 | 19 | 327 | 0.7449 |
| 11-12 | 25 | 352 | 0.8018 |
| 13-14 | 14 | 366 | 0.8337 |
| 15-16 | 10 | 376 | 0.8565 |
| 17-18 | 12 | 388 | 0.8838 |
| 19-20 | 10 | 398 | 0.9066 |
| 21-22 | 13 | 411 | 0.9362 |
| 23-24 | 6 | 417 | 0.9499 |
| 25-26 | 4 | 421 | 0.9590 |
| 27-28 | 3 | 424 | 0.9658 |
| 29-30 | 3 | 427 | 0.9727 |
| 31-35 | 3 | 430 | 0.9795 |
| 36-40 | 3 | 433 | 0.9863 |
| 41-50 | 1 | 434 | 0.9886 |
| 51-60 | 3 | 437 | 0.9954 |
| 61-70 | 1 | 438 | 0.9977 |
| 71-100 | 1 | 439 | 1.0000 |


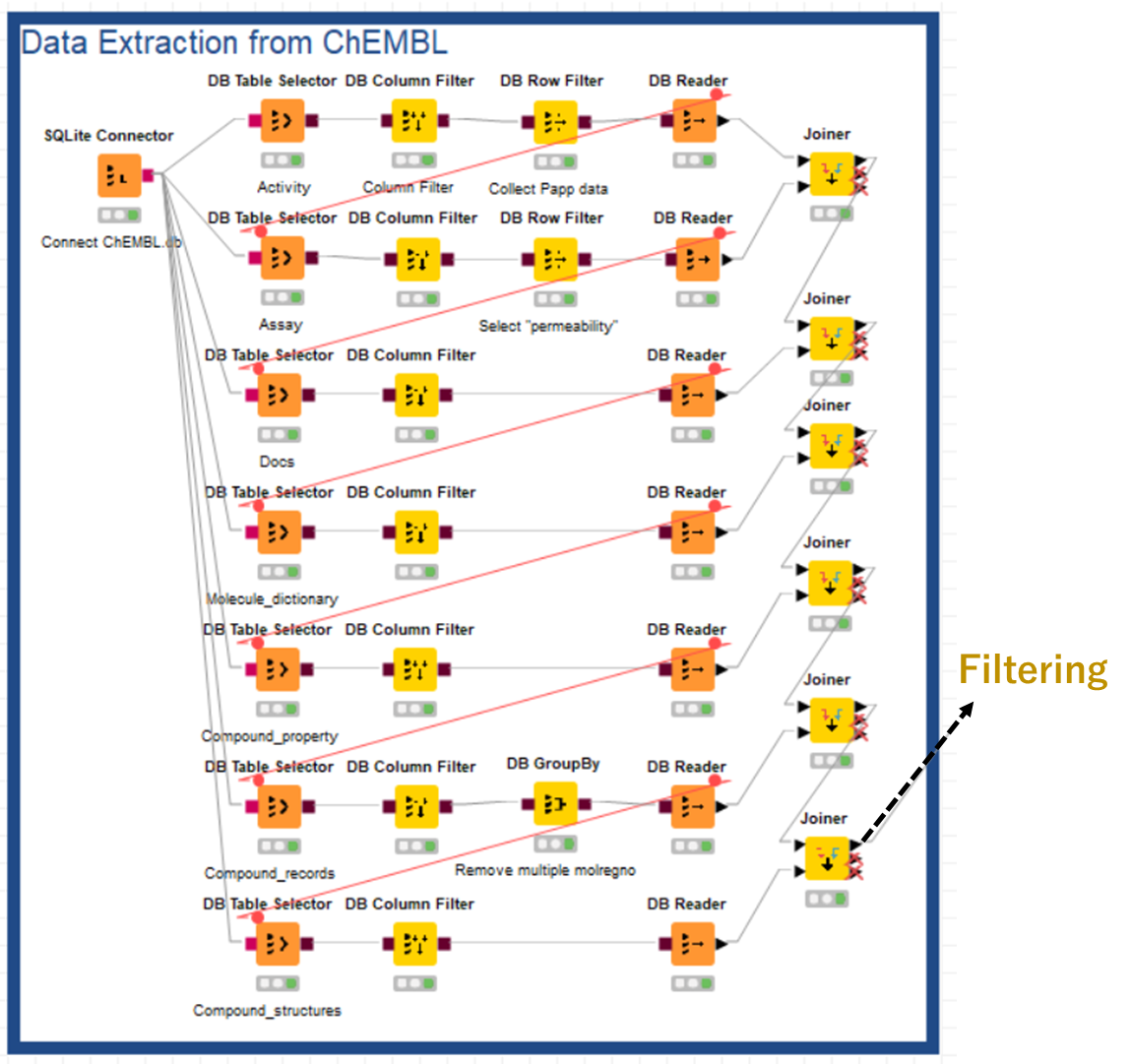


Fig S1. Schema of phase 1: data extraction from ChEMBL. The downloaded ChEMBL SQL db (ver. 28) was imported into KNIME using the SQLite data source connector. Seven data tables (activities, assays, docs, molecule_dictionary, compound_properties, compound_records, and compound_structure) were collected and merged into one table for phase 2.


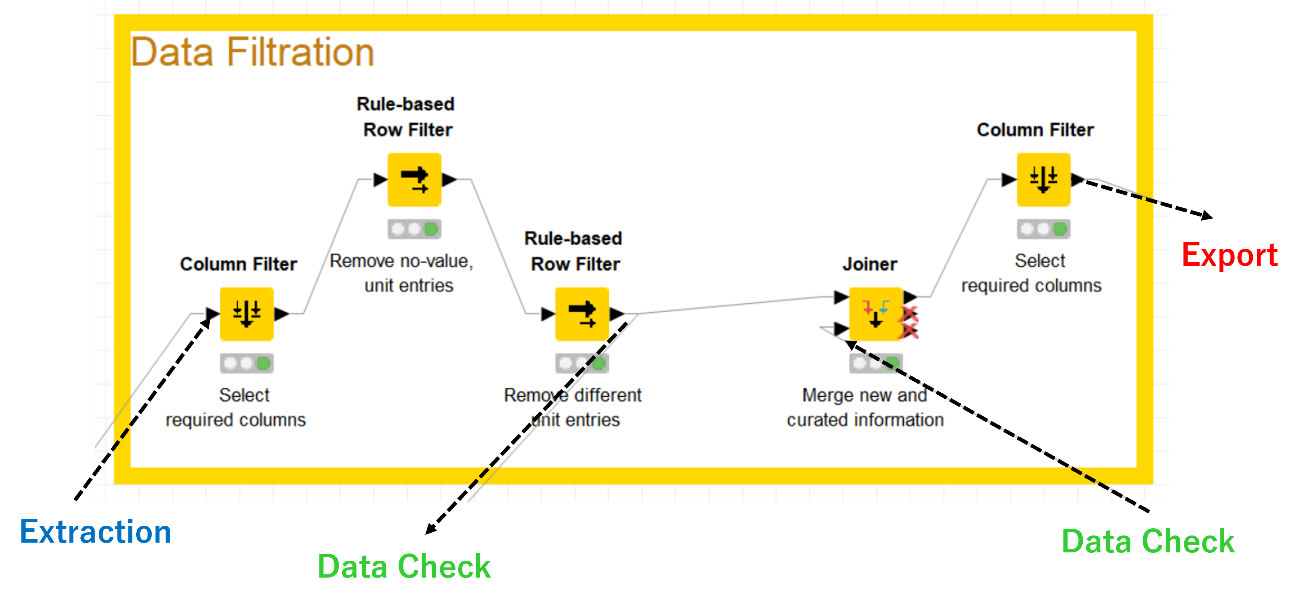


Fig S2. Schema of phase 2: data filtration. The extracted entries were filtered based on the experimental information. The columns used in the table merge (doc_id and assay_type) were removed because they were not used after the previous procedures. The retained entries were passed to the Data Check phase (phase 3). The checked entries were reconnected and moved to the export phase (phase 4).


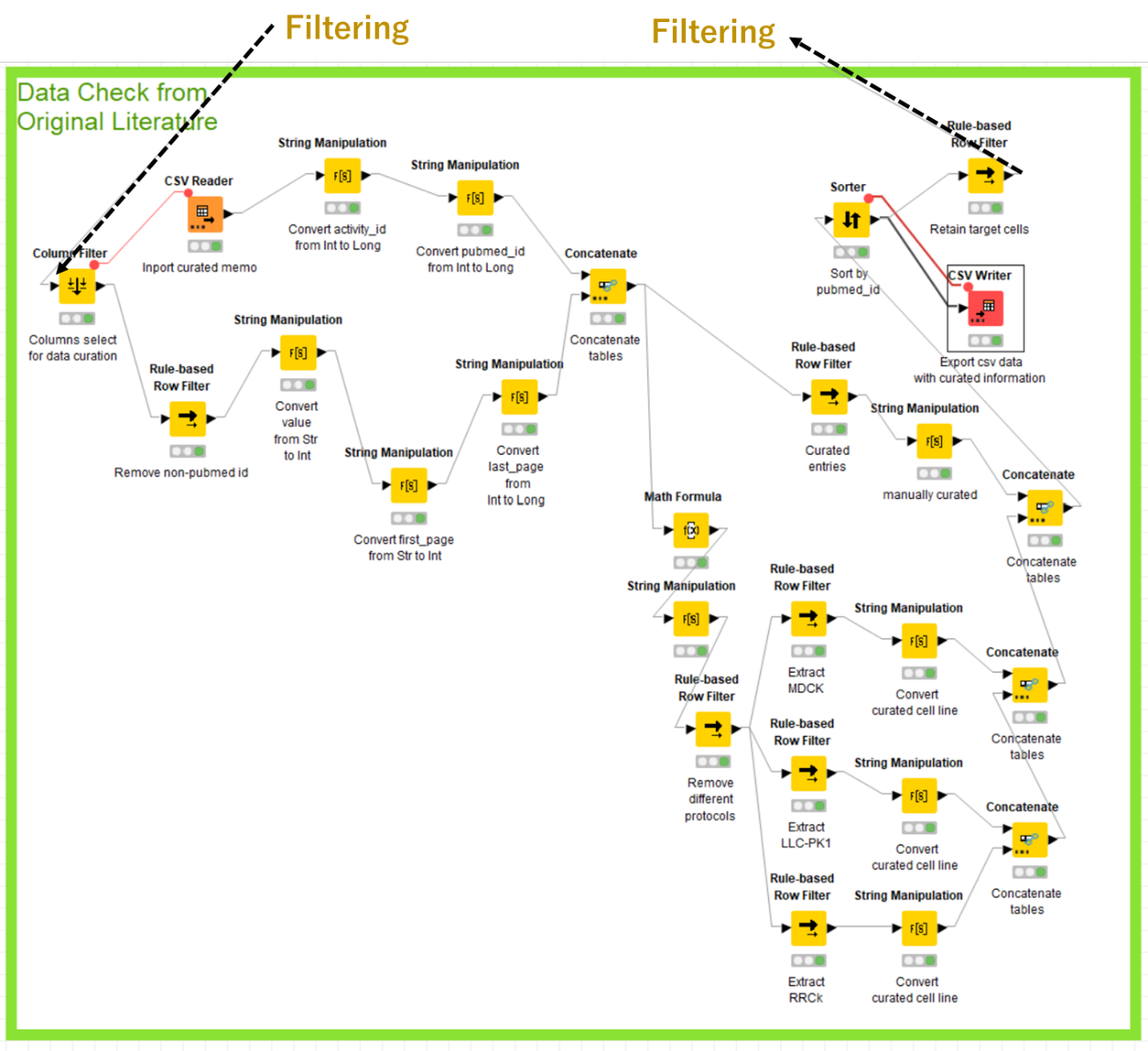


Fig S3. Schema of phase 3: data checking from the original literature. The entries that were measured using the required protocols were retained and moved back to phase 2. The entries measured using different protocols were removed. The checked information from the original articles, and a curated memo was generated during this phase. Initially, entries that were unchecked were collected and compared with previously checked entries. Subsequently, entries requiring checking were outputted from the KNIME workflow to check against the original data. After that, the description of ChEMBL was checked and the entries without MDR1 overexpression and with across the apical to basolateral using MDCK, LLC-PK1 or RRCK were retained. Finally, the articles in these entries were reviewed and the checked results were imported into KNIME and merged with the previously checked entries.

This phase checks the new entries compared to previously checked articles. The previously checked articles were manually checked by authors and the measurement protocols and the used cell lines were included in the csv file, "Check_Papp.csv" which was imported in the CSV Reader node. The protocols and cell lines checked by reading articles were memorized in the "curated_cell_line" column in the csv file. The differences observed when running this workflow on the old and new versions of ChEMBL represent the new data identified by the workflow. This comparison highlights the workflow's efficacy in detecting variations between different database versions. The differences observed when running this workflow on the old and new versions of ChEMBL represent the new data identified by the workflow. This comparison highlights the workflow's efficacy in detecting variations between different database versions. This information is crucial to validate the new data. It serves as an important step in distinguishing between verified and unverified data, facilitating further investigation.


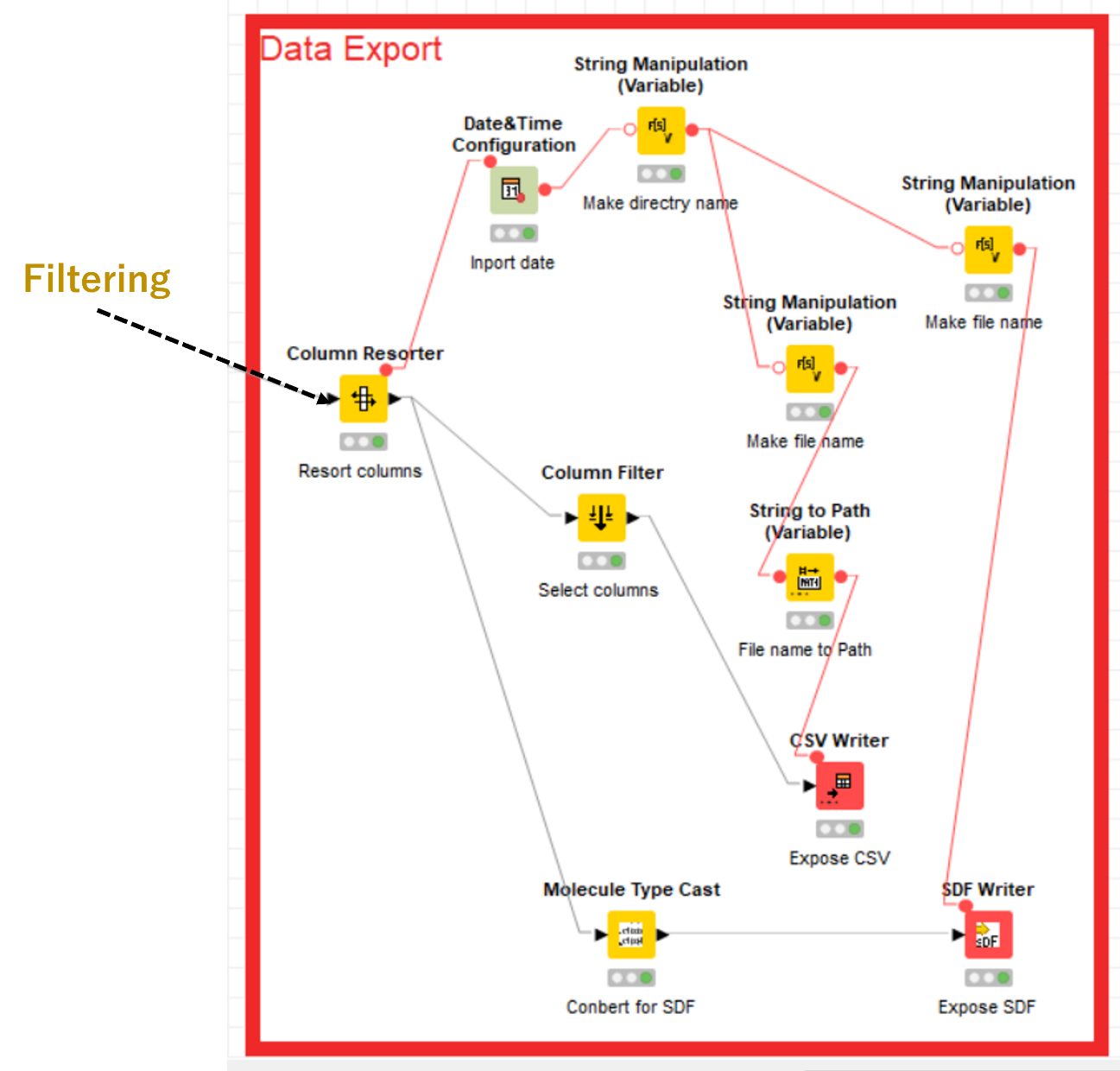


Fig S4. Schema of phase 4: data export. The entries retained through the protocol check were exported to formats that could be used by researchers. Given the variety of tools available, the CSV and SDF formats are convenient.


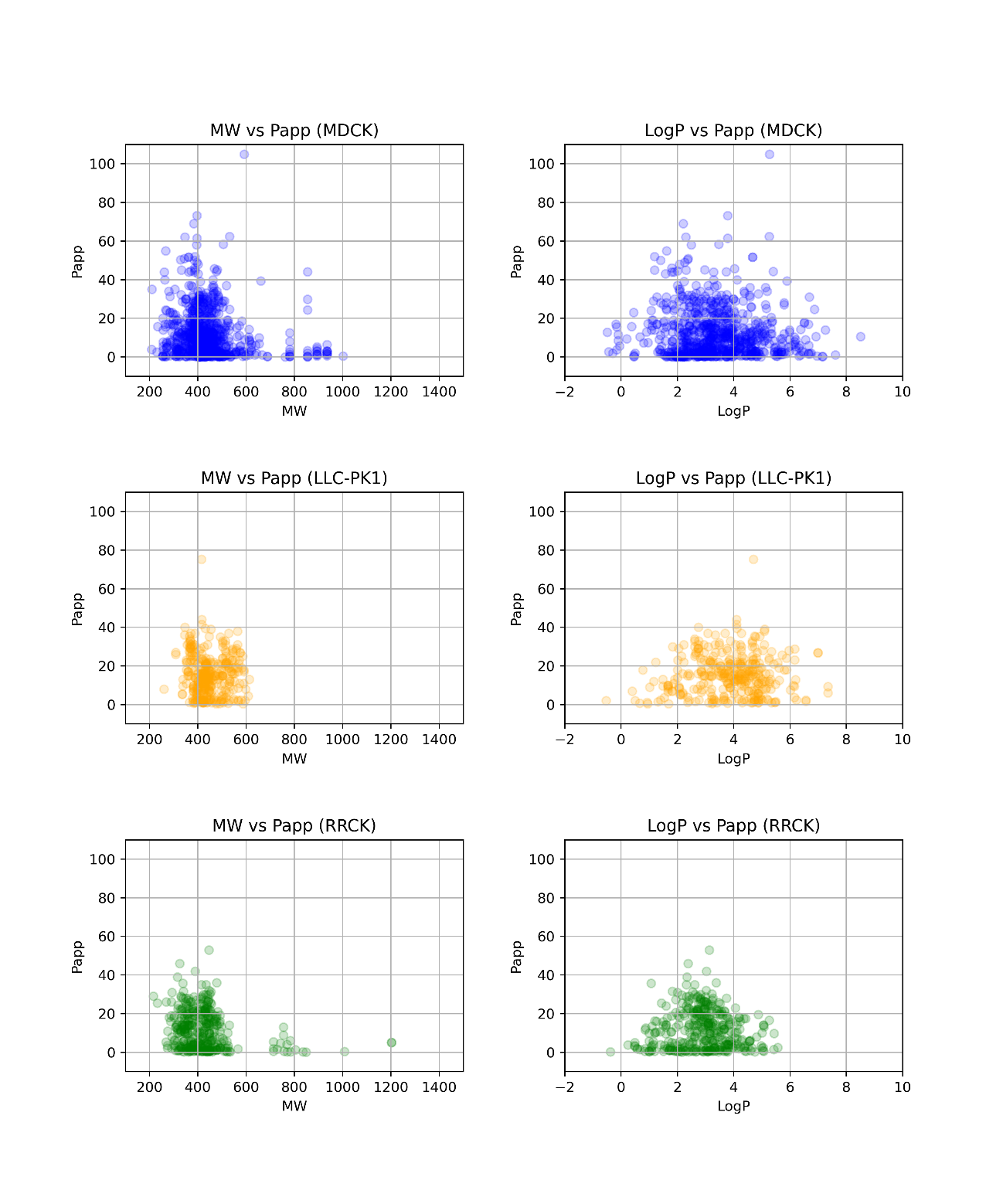


Fig S5. Relationships between features (molecular weight (MW) and LogP) of manually curated compounds. LogP was calculated using RDKit. The left side of the scatter plots shows the relationship between MW and Papp. The right side of the scatter plots show the relationship between LogP and Papp. The upper two scatter plots with blue points represent compounds in the curated MDCK cell dataset. The middle two scatter plots with orange points represent compounds in the curated LLC-PK1 cell dataset. The lower two scatter plots with green points represent compounds in the curated RRCK cell dataset.
